# National survey and point prevalence study of sedation practice in UK critical care

**DOI:** 10.1186/s13054-016-1532-x

**Published:** 2016-10-27

**Authors:** Alvin Richards-Belle, Ruth R. Canter, G. Sarah Power, Emily J. Robinson, Henrik Reschreiter, Hannah Wunsch, Sheila E. Harvey

**Affiliations:** 1 Intensive Care National Audit & Research Centre (ICNARC), Napier House, 24 High Holborn, London, WC1V 6AZ UK; 2 Poole Hospital NHS Foundation Trust, Longfleet Road, Poole, BH15 2JB UK; 3 Department of Critical Care Medicine, Sunnybrook Health Sciences Centre, University of Toronto, 2075 Bayview Avenue, Toronto, ON Canada

**Keywords:** Sedation, Analgesia, Survey, Point prevalence, Critical care

## Abstract

**Background:**

The present study was designed to (1) establish current sedation practice in UK critical care to inform evidence synthesis and potential future primary research and (2) to compare practice reported via a survey with actual practice assessed in a point prevalence study (PPS).

**Methods:**

UK adult general critical care units were invited to participate in a survey of current sedation practice, and a representative sample of units was invited to participate in a PPS of sedation practice at the patient level. Survey responses were compared with PPS data where both were available.

**Results:**

Survey responses were received from 214 (91 %) of 235 eligible critical care units. Of these respondents, 57 % reported having a written sedation protocol, 94 % having a policy of daily sedation holds and 94 % using a sedation scale to assess depth of sedation. In the PPS, across units reporting a policy of daily sedation holds, a median of 50 % (IQR 33–75 %) of sedated patients were considered for a sedation hold. A median of 88 % (IQR 63–100 %) of patients were assessed using the same sedation scale as reported in the survey. Both the survey and the PPS indicated propofol as the preferred sedative and alfentanil, fentanyl and morphine as the preferred analgesics. In most of the PPS units, all patients had received the unit’s reported first-choice sedative (median across units 100 %, IQR 64–100 %), and a median of 80 % (IQR 67–100 %) of patients had received the unit’s reported first-choice analgesic. Most units (83 %) reported in the survey that sedatives are usually administered in combination with analgesics. Across units that participated in the PPS, 69 % of patients had received a combination of agents – most frequently propofol combined with either alfentanil or fentanyl.

**Conclusions:**

Clinical practice reported in the national survey did not accurately reflect actual clinical practice at the patient level observed in the PPS. Employing a mixed methods approach provided a more complete picture of sedation practice in terms of breadth and depth of information.

**Electronic supplementary material:**

The online version of this article (doi:10.1186/s13054-016-1532-x) contains supplementary material, which is available to authorized users.

## Background

Sedation and analgesia are frequently administered to patients in critical care units to facilitate therapies, such as mechanical ventilation and other invasive procedures, with the objectives of ensuring patient safety, comfort and sometimes amnesia. Commonly used sedatives are propofol, benzodiazepines and alpha-2 agonists (clonidine and dexmedetomidine). Guidelines favour sedation strategies using agents such as propofol or dexmedetomidine over strategies using benzodiazepines to improve clinical outcomes in mechanically ventilated patients [1]. Previous surveys conducted in the United Kingdom (UK) [2, 3] and elsewhere [4] indicate a shift away from benzodiazepines toward propofol for sedating patients in critical care. Guidelines on sedation also include recommendations for pain management, with an emphasis on assessing and treating pain first before administering sedative medication [1].

The Intensive Care National Audit & Research Centre (ICNARC) was commissioned by the National Institute for Health Research Health Technology Assessment Programme to design and conduct a study to establish current sedation practice in UK adult critical care. The overall aim of the study was to provide baseline data on current practice to inform evidence synthesis and potential future primary research. Specific objectives were to establish (1) through a survey of all UK adult general critical care units, reported current sedation practice; and (2) through a point prevalence study (PPS) in a representative sample of UK adult general critical care units, the current prevalence of use of sedative agents and regimens. This combination of study designs was selected to give the most complete picture of current practice in terms of both the breadth and the depth of information and to examine reported versus actual clinical practice.

## Methods

### National survey

National Health Service (NHS) adult general critical care units were identified from databases maintained by ICNARC, and the Scottish Intensive Care Society Audit Group [5]. A general critical care unit was defined as an intensive care unit (ICU) or combined ICU-high-dependency unit (HDU). Stand-alone HDUs and speciality units (e.g., neurosciences, cardiothoracic) were excluded.

A review of previous surveys [2, 3, 6–10] informed development of the survey tool (Additional file 1: Figure S1), which captured information on management of sedation as well as the use of specific sedative and analgesic agents. With respect to analgesia, the focus was on analgesic agents with a sedative effect, such as intravenous opioids, rather than on oral or regional analgesia. Following piloting, the survey was sent via email, in January 2014, to the clinical directors of all UK adult general critical care units, who were asked to complete the survey either online (via SurveyMonkey®), electronically or on paper. Non-responders were followed up, with a second email and then a telephone call, until May 2014.

### Point prevalence study

The PPS was nested in the Case Mix Programme (CMP), the national comparative clinical audit for adult critical care in England, Wales and Northern Ireland, coordinated by ICNARC. At the time, 95 % of all possible adult general critical care units were participating in the CMP.

It was estimated that a sample size of 50 units would be required to give representation in terms of unit characteristics and to allow for variation in sedation practice. Based on CMP data, the mean number of patients in an average-sized unit at 1400 on a midweek day is 9; therefore, the projected total sample size was 450 patients, of whom it was anticipated that approximately 45 % (200 patients) would be sedated. Because the aim was to recruit 50 units, the invitation to take part was restricted to 97 actively participating units that were the most up-to-date with data submission and validation at the time. Those that agreed to take part were asked to complete a short data collection form (Additional file 2: Figure S2) for every patient in the unit at 1400 on 11 December 2013. For sedated patients (i.e., those who had received a sedative and/or analgesic in the previous 24 h), staff were asked to provide information on management of sedation as well as sedative and/or analgesic agents received. Completed data collection forms were returned either electronically or on paper.

### Statistical analysis

For the analyses, we used data from the CMP database, which contains pooled case mix and outcome data on consecutive admissions to participating units and has been independently assessed to be of high quality [11]. Responses to categorical questions in the survey were summarised as the number (percentage) of respondents selecting each response. Continuous data were summarised as the mean, SD and range (minimum to maximum) across the responses.

Patients in the PPS were compared with all patients in CMP adult general critical care units at 1400 on 11 December 2013 for case mix, outcome and length of stay. Numbers (percentages) of sedated patients assessed using a sedation scale/score were summarised in addition to, among patients who had been in the unit for at least 24 h, those considered for and/or receiving a sedation hold during the previous 24 h. The number (percentage) of patients receiving each sedative and analgesic and combinations of agents during the previous 24 h were summarised together with, for agents received by five or more patients, the mean (SD) of the highest rate of infusion (in milligrams per hour) and the total dose (in milligrams), including both infusions and boluses. With respect to analgesia, analgesia administered orally or regionally (e.g., epidural) was excluded from the analysis.

Survey responses were compared with PPS data, where both were available, to investigate the degree to which reported practice reflected actual practice. For units that reported daily sedation holds, the number (percentage) of sedated patients who had been in the unit for at least 24 h and who had been considered for a sedation hold was summarised. Across all units, the median proportion and IQR of sedated patients considered for a sedation hold were calculated.

For each sedation scale/score reported, the number (percentage) of sedated patients in the PPS who were assessed using the reported scale/score was summarised. The median (IQR) proportion of patients assessed using the same sedation scale/score as reported was calculated across all units. For patients who had received a sedative agent, the proportion that received the unit’s reported first-choice agent was summarised. The median (IQR) proportion of patients receiving any sedative agent who received the reported first-choice agent was calculated across all units. The same approach was repeated for analgesic agents.

All data analyses were conducted using Stata/SE version 13.0 software (StataCorp LP, College Station, TX, USA). Support for the collection and use of patient-identifiable data without consent for the CMP was obtained under section 251 of the NHS Act 2006 (approval PIAG 2-10(f)/2005).

## Results

### National survey

Of 235 eligible NHS adult general critical care units, completed surveys were received from 214 (91.1 %). Response rates were similar across different countries (apart from Wales, in which the response rate was lower), hospital types, unit sizes and participation or not in the CMP (Additional file 3: Table S1). The majority of survey responses were from the clinical director/lead clinician (40.2 %) or another medical consultant in the unit (31.3 %).

More than half (*n* = 122, 57.0 %) of units reported having a written sedation or sedation/analgesia protocol. Of these, 26 units (23.0 %) reported that compliance was routinely audited; the approximate level of compliance reported was a mean of 65 % (SD 20 %, range 26–100 %). Almost all units (93.9 %) reported routinely using a sedation scale/score to assess the depth of sedation (Additional file 4: Table S2). Of these, approximately two-thirds (64.7 %) reported using the Richmond Agitation-Sedation Scale (RASS) [12] and a quarter (24.9 %) reported using the Ramsay Sedation Scale (RSS) [13]. Similarly, almost all units (93.9 %) reported that a sedation hold was considered daily for patients. Of these, 95 (47.3 %) units reported routinely auditing compliance; the approximate level of compliance reported was a mean of 82 % (SD 18 %, range 30–100 %) across the 89 units that provided this information. Of the 149 units (69.6 %) that reported daily screening for delirium, almost all (93.3 %) reported using the Confusion Assessment Method for the Intensive Care Unit (CAM-ICU) [14].

Propofol was the most popular sedative agent; 98.1 % of units reported very frequent/frequent use (Table 1). Around one-third (32.2 %) of units reported very frequent/frequent use of midazolam. Of the alpha-2 agonists, very frequent/frequent use of clonidine was reported by 32.7 % of units, compared with 10.3 % for use of dexmedetomidine. A high percentage of units (88.3 %) reported that propofol was generally their first choice of sedative agent, compared with only 6.1 % reporting midazolam (three units reported both agents) (Additional file 5: Table S3).

**Table 1:** National survey: sedative agents, analgesic agents, sedative/analgesic delivery regimens and their reported frequency of use

	Reported frequency of use, *n* (%)			
	Very frequently/frequently	Occasionally/rarely	Never	Not reported
Sedative agent				
Propofol	210 (98.1)	2 (0.9)	0 (0)	2 (0.9)
Midazolam	69 (32.2)	130 (60.7)	11 (5.1)	4 (1.9)
Diazepam	4 (1.9)	96 (44.9)	105 (49.1)	9 (4.2)
Lorazepam	2 (0.9)	120 (56.1)	83 (38.8)	9 (4.2)
Clonidine	70 (32.7)	129 (60.3)	8 (3.7)	7 (3.3)
Dexmedetomidine	22 (10.3)	57 (26.6)	127 (59.3)	8 (3.7)
Haloperidol	79 (36.9)	115 (53.7)	13 (6.1)	7 (3.3)
Atypical anti-psychotic^a^	15 (7.0)	94 (43.9)	89 (41.6)	16 (7.5)
Other	26 (12.1)	12 (5.6)	–	176 (82.2)
Analgesic agent				
Morphine	90 (42.1)	114 (53.3)	6 (2.8)	4 (1.9)
Fentanyl	77 (36.0)	89 (41.6)	41 (19.2)	7 (3.3)
Alfentanil	110 (51.4)	53 (24.8)	46 (21.5)	5 (2.3)
Remifentanil	72 (33.6)	106 (49.5)	32 (15.0)	4 (1.9)
Ketamine	1 (0.5)	177 (82.7)	28 (13.1)	8 (3.7)
Other	3 (1.4)	9 (4.2)	–	202 (94.4)
Sedative/analgesic delivery regimen				
Single sedative agent	47 (22.0)	142 (66.4)	4 (1.9)	21 (9.8)
Sedative(s) in combination with one or more analgesic agents	207 (96.7)	4 (1.9)	0 (0)	3 (1.4)
Multiple sedatives together	23 (10.8)	163 (76.2)	5 (2.3)	23 (10.7)

^a^ For sedative purposes only

Alfentanil and morphine were the most popular analgesic agents; 51.5 % and 42.1 % of units, respectively, reported very frequent/frequent use (Table 1). Most units (39.7 %) reported that generally alfentanil was their first choice of analgesic agent, with around one-fourth (26.2 %) reporting fentanyl (Additional file 6: Table S4).

Nearly all units (96.7 %) reported that they very frequently/frequently deliver sedative(s) in combination with analgesic(s) (Table 1), with the majority (82.7 %) reporting this to be their first choice for delivery of sedative and analgesic regimens (Additional file 7: Table S5). Although cost was reported by nearly half (47.7 %) of units as very important/important in determining the choice of sedative and/or analgesic agent, 82.2 % of units reported that the expected duration of sedation/analgesia was very important/important (Additional file 8: Table S6).

### Point prevalence study

Fifty-two units participated, and validated CMP data were available for 516 patients in 50 units. In addition, validated CMP data were available for 1296 patients in 133 non-participating CMP units on the day of the study. In general, unit characteristics (Additional file 9: Table S7) and the case mix and outcomes of patients (Additional file 10: Table S8, Additional file 11: Table S9) were similar for participating and non-participating PPS units.

Fifty-one units participated in both the PPS and the national survey. Sedation practices reported by these units were similar to sedation practices reported by the 163 units that participated in the national survey only (Additional file 12: Table S10, Additional file 13: Table S11, Additional file 14: Table S12, Additional file 15: Table S13, Additional file 16: Table S14).

Of the 550 patients in the 52 participating units on the study day, a completed data collection form was returned for 541 patients (98.4 %), 242 (44.7 %) of whom had been sedated during the previous 24 h. Of the 541 patients with a completed data collection form, validated CMP data were available for 516 patients (95.4 %) (*n* = 230 sedated patients), enabling comparison of characteristics and outcomes of sedated and non-sedated patients. Sedated patients were slightly younger than non-sedated patients (mean age 59.3 years versus 63.6 years) but similar in terms of gender, ethnicity and severe co-morbidities (Additional file 17: Table S15). Sedated patients had higher acute severity of illness and were more likely to have been mechanically ventilated (78.4 % versus 43.7 %) during the first 24 h in the unit, and had, on average, been in the unit for a shorter time at the point of the study (mean 6.9 days versus 10.6 days). On the day of the study, 12.0 % (*n* = 29) of the sedated patients were being ventilated via a tracheostomy.

Of the 242 sedated patients in the 52 participating units, 34 (14.0 %) had received only sedatives, 42 (17.4 %) only analgesics and 166 (68.6 %) both sedative and analgesic agents. A sedation scale/score was recorded for 222 patients (91.7 %). Of these, two-thirds (66.7 %) were assessed using the RASS [12], with the remaining patients assessed using the RSS [13] (20.3 %) or other scales (13.1 %). Of patients eligible for a sedation hold (*n* = 186), 100 (53.8 %) had been assessed, and 77 (77.0 %) had received a sedation hold during the previous 24 h.

The most frequently used sedative was propofol, received by over two-thirds (71.9 %) of patients during the previous 24 h, with midazolam and clonidine received by 21 (8.7 %) and 20 (8.3 %) patients, respectively (Table 2). Use of analgesic agents was more variable; alfentanil and fentanyl were the most frequently used agents (31.4 % and 27.3 %, respectively). The most common combinations of sedative and analgesic agents were propofol combined with either alfentanil (*n* = 49, 20.3 %) or fentanyl (*n* = 44, 18.2 %) (Table 3). In isolation, propofol was the most frequently used sedative (*n* = 15, 6.2 %) and morphine the most frequently used analgesic (*n* = 22, 9.1 %).

**Table 2:** Point prevalence study: sedative and analgesic agents received

	Received	Highest rate of infusion (mg/h)		Total dose (mg)	
	*n* (%)	Reported, *n*	Mean (SD)^a^	Reported, *n*	Mean (SD)^a^
Sedative agent					
Propofol	174 (71.9)	162	138 (87)	172	2052 (1734)
Haloperidol	8 (3.3)	1	–	8	6.6 (3.8)
Midazolam	21 (8.7)	16	7.1 (4.8)	21	97.2 (121.5)
Clonidine	20 (8.3)	9	0.17 (0.18)	18	1.3 (1.9)
Dexmedetomidine	4 (1.7)	3	–	3	–
Ketamine	1 (0.4)	1	–	1	–
Diazepam	4 (1.7)	0	–	4	–
Lorazepam	3 (1.2)	0	–	2	–
Olanzapine	2 (0.8)	0	–	1	–
Thiopentone	2 (0.8)	0	–	2	–
Analgesic agent					
Alfentanil	76 (31.4)	74	2.3 (1.9)	76	40.9 (36.6)
Fentanyl	66 (27.3)	59	0.15 (0.21)	59	1.7 (1.5)
Remifentanil	24 (9.9)	22	0.57 (0.63)	21	9.3 (9.6)
Morphine	48 (19.8)	20	4.3 (4.5)	46	57.8 (83.8)

^a^ Included only if reported for at least five patients

**Table 3:** Point prevalence study: most frequent combinations of sedative and analgesic agents and single sedative/analgesic agents

Sedative/analgesic agents	*n* (%)
Most frequent^a^ combinations of sedative and analgesic^b^ agents	
Propofol and alfentanil	49 (20.3)
Propofol and fentanyl	44 (18.2)
Propofol and remifentanil	12 (5.0)
Propofol and morphine	10 (4.1)
Propofol, midazolam and alfentanil	4 (1.7)
Propofol, midazolam and fentanyl	4 (1.7)
Propofol, clonidine and alfentanil	3 (1.2)
Most frequent^a^ single sedative agents	
Propofol	15 (6.2)
Clonidine	5 (2.1)
Most frequent^a^ single analgesic^b^ agents	
Morphine	22 (9.1)
Fentanyl	8 (3.3)
Alfentanil	5 (2.1)
Remifentanil	4 (1.7)

^a^ Combinations/single agents included only if reported for at least three patients

^b^ With or without additional paracetamol, codeine or bupivacaine

### Comparison of reported versus actual practice

In units that reported in the national survey that a sedation hold is considered daily for patients, just over half (*n* = 97, 53.0 %) of eligible patients in the PPS had been considered for a sedation hold in the previous 24 h. Across units, the median percentage of patients who had been considered for a sedation hold was 50 % (IQR 33–75 %).

Across units, a median of 88 % (IQR 63–100 %) of patients were assessed using the same sedation scale/score as reported (Table 4). Two units that reported using the RSS [13] had assessed the majority of patients in the PPS using the RASS [12], and two units that reported using the RASS had assessed the majority of patients using the RSS, suggesting possible confusion between these scales with similar names.

**Table 4:** Reported versus actual sedation scale/score use

Point prevalence study	Sedation scale/score reported in national survey		
	Richmond Agitation-Sedation Scale	Ramsay Sedation Scale	Riker Sedation-Agitation Scale
Patients who received any sedative/analgesic agent^a^, *n*	149	56	12
Sedation scale/score used, *n* (%)			
Assessed with reported scale/score	114 (76.5)	27 (48.2)	11 (91.7)
Assessed with different scale/score	26 (17.4)	10 (17.9)	0 (0)
Not assessed with a scale/score	9 (6.0)	19 (33.9)	1 (8.3)
Percentage of patients assessed with reported scale/score across units^b^, median (IQR)	88 (63–100)		

^a^ Sedated patients in units that participated in the point prevalence study and reported using the indicated sedation scale/score

^b^ Excluding units with fewer than three eligible patients (*n* = 20)

In units that reported propofol as their first-choice sedative, the majority (87.6 %) of sedated patients had received propofol (Table 5). However, in units that indicated midazolam as their first choice, only one-third (33.3 %) of patients had received midazolam. Actual use of the reported first-choice analgesic varied from 93.4 % among patients in units that had reported fentanyl as their first choice to 53.4 % among patients in units that had reported morphine as their first choice. Across units, a median of 80 % (IQR 67–100 %) of patients received the unit’s reported first-choice analgesic.

**Table 5:** Reported versus actual sedative and analgesic agent use

Point prevalence study	First-choice sedative agent reported in national survey			
	Propofol		Midazolam	
Patients who received any sedative agent^a^, *n*	170		18	
Patients who received unit’s first-choice sedative agent, *n* (%)	149 (87.6)		6 (33.3)	
Percentage of patients who received first-choice sedative across units^b^, median (IQR)	100 (64–100)			
Point prevalence study	First-choice analgesic agent reported in national survey^c^			
	Alfentanil	Fentanyl	Remifentanil	Morphine
Patients who received any analgesic agent^d^, *n*	75	61	22	28
Patients who received unit’s first-choice analgesic agent, *n* (%)	58 (77.3)	57 (93.4)	12 (54.5)	15 (53.4)
Percentage of patients who received first-choice analgesic across units^e^, median (IQR)	80 (67–100)			

^a^ In critical care units reporting in the national survey that they use the indicated sedative agent as their first choice

^b^ Excluding units with fewer than three eligible patients (*n* = 23)

^c^ Ten patients in two units that reported using two alternative analgesic agents as their first choice are excluded from data for individual agents but included in summary data across units (where either of the two indicated agents may have been used)

^d^ In critical care units reporting in the national survey that they use the indicated analgesic agent as their first choice

^e^ Excluding units with fewer than three eligible patients (*n* = 19)

## Discussion

Of the 235 UK NHS adult general critical care units identified, a high proportion (91.1 %) responded to the survey. In addition, a representative sample of 52 units also participated in the PPS. Data derived from the two studies indicated that *reported* practice does not necessarily reflect *actual* practice. Most (88 %) PPS patients were assessed using the same sedation scale/score reported in the survey; however, there was some variation across units. Furthermore, although a high proportion (94 %) of units reported using daily sedation holds, overall only half of sedated patients in the PPS who had been in the unit for 24 h or more had been considered for a sedation hold during the previous 24 h.

Both the survey and PPS indicated that propofol is the preferred sedative and alfentanil, fentanyl and morphine the preferred analgesics in UK critical care. Most units (83 %) reported frequently/very frequently administering sedatives in combination with analgesics, and around two-thirds (68.6 %) of patients in the PPS had received a combination of sedatives and analgesics, most frequently propofol combined with either alfentanil or fentanyl.

The use of guidelines or protocols for management of pain, agitation and delirium is strongly recommended [1, 15]. Only 57 % of units reported having a written sedation protocol, similar to the findings of a recent Internet-based survey of UK critical care pharmacists in which 55 % of respondents reported use of sedation guidelines [16]. However, this is considerably lower than previously reported in the United Kingdom. In a telephone survey conducted in 2011 [17] and a postal survey [3] conducted in 2007, 82 % of units reported having a written sedation policy and 80 % having a sedation guideline, respectively. The different responses could be related to how the question was phrased and the mode of administration. The rate of protocol implementation reported in our survey is similar to rates that have been reported in other countries, including Australia (54 %) [18], Germany (52 %) [8] and the United States (64 %) [19].

The RASS [12] is recommended [1] as one of the most valid and reliable subjective sedation scales for measuring depth of sedation [20]. The reported use of this sedation scale has increased considerably in the UK since 2007 [3], from 5 % to 65 % of units, with a general shift away from using the RSS [13]; a quarter of units reported using this scale/score, compared with 67 % in 2007 [3].

The practice of daily sedation holds is recommended [1, 15, 21] and has been incorporated into the ventilator care bundle in the UK [22]. The benefits of minimising sedation include less time on a mechanical ventilator, fewer complications and reduced length of stay in critical care [23–25]. The proportion of UK units that reported practicing daily sedation holds was higher (94 %) than previously reported in 2007 (78 %) [3] and more recently in 2014 (80 %) [16]. Data derived from the PPS suggest that overall compliance is possibly much lower. Of the patients who had been in the unit for at least 24 h, around half (53 %) had been considered for a sedation hold in the previous 24 h, and of these, 77 % had their sedation withheld.

Previous surveys in the UK [2, 3, 16] and elsewhere [4] indicate a shift from benzodiazepines to propofol for sedating patients. Both the survey and the PPS indicated that propofol is by far the most widely used sedative agent in the UK. Although around one-third of units reported using midazolam, very few reported it to be their first choice. Use of the alpha-2 agonist clonidine has increased in the UK, with around one-third of units reporting very frequent/frequent use, whereas use of dexmedetomidine is rare. It seems that uptake of dexmedetomidine in UK critical care has been slow since it was licensed for use in 2011. Cost may be a factor, with clinicians preferring to use established and often cheaper alternatives.

Intravenous opioids are recommended as first-line agents for non-neuropathic pain in critically ill patients. Authors of previous UK surveys [2, 3] have reported that alfentanil, fentanyl and morphine are the most frequently used opioids. Our findings are similar, although both the survey and the PPS suggest a trend away from morphine toward agents such as alfentanil and fentanyl as the first choice for analgesia. Authors of surveys done elsewhere have reported a similar trend, although morphine is still widely used [4]. Even so, authors of a recent survey of UK critical care pharmacists reported morphine to be the most common first-line prescription in almost half (49 %) of units [16].

Surveys are frequently used for establishing current clinical practice in a variety of healthcare settings, their advantage being that they are relatively cheap, quick and simple to conduct, although achieving high response rates can be challenging. A high response rate (91 %) was achieved in our survey, comparing favourably with some previous UK surveys with response rates of 79 % [2], 64 % [3], 78 % [17] and 60 % [16]. Elsewhere, response rates have ranged from 20 % [9] to 84 % [7]. One limitation of surveys, however, is that reported practice does not necessarily reflect the reality of actual clinical practice at the patient level. A major strength of the present study is the combination of a national survey of adult general critical care units and a PPS, which enabled us to examine both *reported* sedation practice across the UK and *actual* practice at the patient level in a representative sample of UK units. We were unable to investigate the reasons for the discrepancies observed between reported practice and actual practice because of limited resources for the study. However, it is likely that these reasons are multifactorial. For example, there may be a lag between a unit policy being initiated and staff at the bedside changing their clinical practice, or the discrepancy may reflect the difficulties in applying universal policies on sedation and analgesia to individual patients, such as that the choice of agent depends on a number of individual patient factors. Another possible explanation is that the person completing the survey is influenced by her own practice when reporting unit practice, which may not necessarily reflect that of her colleagues or may not even be in line with the unit policy. Our results indicate a need for improved auditing of clinical practice within units to ensure that agreed unit policy on clinical practice is being followed and, if not, to identify the reasons. Less than half of units reported in the survey that they audit compliance with policies, such as sedation holds, and among those that did, there was considerable variability in compliance with unit policy.

A possible limitation of the present study is that the PPS and the survey were not conducted contemporaneously. However, both were completed within a 5-month period, and it seems unlikely that during this time there were any major changes in policy within units that might explain the discrepancies in clinical practice observed. Furthermore, a potential strength of not conducting the two studies contemporaneously is that the PPS provided a snapshot of actual clinical practice before units were asked about their clinical practice in the survey. Nesting the PPS within the CMP allowed us to conduct a detailed examination of case mix and outcomes of patients who were in a CMP unit on the day of the study. Patient populations in CMP units that did and did not participate in the PPS were very similar with respect to case mix and outcomes. Furthermore, comparison of the survey data from 51 units that participated in the PPS with all other responding units did not reveal any differences in reported sedation practices, suggesting that units that participated in the PPS were a representative sample.

A PPS is relatively quick and simple to conduct. However, a limitation is that it will only provide a snapshot of clinical practice at a single point in time. It is also more likely to capture those patients who stay longer in the critical care unit. Sedation and analgesia regimens will vary according to the expected duration of sedation and analgesia, which is an important factor in determining the choice of agents.

## Conclusions

This study demonstrates that clinical practice reported in the national survey did not accurately reflect actual clinical practice at the patient level. Employing a mixed methods approach provided a more complete picture of current sedation practice in UK critical care in terms of the breadth and the depth of information.

## Additional files

Additional file 1: Figure S1. National survey tool. National survey instrument of sedation practice used in the present study. (PDF 226 kb)
Additional file 2: Figure S2. Point prevalence study data collection form. PPS data collection form used to collect data on all admissions in critical care units participating in the PPS on the study day. (PDF 145 kb)
Additional file 3: Table S1. National survey: unit characteristics by response. (PDF 69 kb)
Additional file 4: Table S2. National survey: sedation scale score in use reported by units. (PDF 61 kb)
Additional file 5: Table S3. National survey: first-choice sedative agent reported by units. (PDF 58 kb)
Additional file 6: Table S4. National survey: first-choice analgesic agent reported by units. (PDF 61 kb)
Additional file 7: Table S5. National survey: first choice of sedative analgesic delivery regimen reported by units. (PDF 59 kb)
Additional file 8: Table S6. National survey: factors determining the choice of sedative/analgesic agents reported by units. (PDF 61 kb)
Additional file 9: Table S7. Characteristics of units in the Case Mix Programme at the time of the point prevalence study (1400 on 11 December 2013) by units that did and did not participate in the point prevalence study. (PDF 10 kb)
Additional file 10: Table S8. Characteristics of patients in Case Mix Programme units at the time of the point prevalence study (1400 on 11 December 2013) by units that did and did not participate in the point prevalence study. (PDF 80 kb)
Additional file 11: Table S9. Outcome and length of stay for patients in Case Mix Programme units on the day of the point prevalence study (1400 on 11 December 2013) by units that did and did not participate in the point prevalence study. (PDF 71 kb)
Additional file 12: Table S10. Sedation score/scale in use reported in the national survey by units that did and did not participate in the point prevalence study. (PDF 7 kb)
Additional file 13: Table S11. Sedative agents, analgesic agents and sedative/analgesic delivery regimens and their reported frequency of use in the national survey by units that did and did not participate in the point prevalence study. (PDF 86 kb)
Additional file 14: Table S12. First choice of sedative agent reported in the national survey by units that did and did not participate in the point prevalence study. (PDF 6 kb)
Additional file 15: Table S13. First choice of analgesic agent reported in the national survey by units that did and did not participate in the point prevalence study. (PDF 7 kb)
Additional file 16: Table S14. First choice for delivery of sedation and analgesia in the national survey by units that did and did not participate in the point prevalence study. (PDF 6 kb)
Additional file 17: Table S15. Point prevalence study: characteristics, outcomes and length of stay of sedated and non-sedated patients with validated Case Mix Programme data (*n* = 50 units). (PDF 86 kb)

## Abbreviations

CAM-ICU: Confusion Assessment Method for the Intensive Care Unit
CMP: Case Mix Programme
HDU: High-dependency unit
ICNARC: Intensive Care National Audit & Research Centre
ICU: Intensive care unit
NHS: National Health Service
PPS: Point prevalence study
RASS: Richmond Agitation-Sedation Scale
RSS: Ramsay Sedation Scale

## References

[1] Barr J, Fraser GL, Puntillo K, Ely EW, Gélinas C, Dasta JF (2013). Clinical practice guidelines for the management of pain, agitation, and delirium in adult patients in the intensive care unit. Crit Care Med.

[2] Murdoch S, Cohen A (2000). Intensive care sedation: a review of current British practice. Intensive Care Med.

[3] Reschreiter H, Maiden M, Kapila A (2008). Sedation practice in the intensive care unit: a UK national survey. Crit Care.

[4] Mehta S, McCullagh I, Burry L (2009). Current sedation practices: lessons learned from international surveys. Crit Care Clin.

[5] Scottish Intensive Care Society Audit Group. Participating units. Available from: http://www.sicsag.scot.nhs.uk/about/participants.html. Accessed 25 Oct 2016.

[6] Patel RP, Gambrell M, Speroff T, Scott TA, Pun BT, Okahashi J (2009). Delirium and sedation in the intensive care unit: survey of behaviors and attitudes of 1384 healthcare professionals. Crit Care Med.

[7] Martin J, Parsch A, Franck M, Wernecke KD, Fischer M, Spies C (2005). Practice of sedation and analgesia in German intensive care units: results of a national survey. Crit Care.

[8] Martin J, Franck M, Sigel S, Weiss M, Spies C (2007). Changes in sedation management in German intensive care units between 2002 and 2006: a national follow-up survey. Crit Care.

[9] Soliman HM, Melot C, Vincent JL (2001). Sedative and analgesic practice in the intensive care unit: the results of a European survey. Br J Anaesth.

[10] Egerod I, Albarran JW, Ring M, Blackwood B (2013). Sedation practice in Nordic and non-Nordic ICUs: a European survey. Nurs Crit Care.

[11] Harrison DA, Brady AR, Rowan K (2004). Case mix, outcome and length of stay for admissions to adult, general critical care units in England, Wales and Northern Ireland: the Intensive Care National Audit & Research Centre Case Mix Programme Database. Crit Care.

[12] Ely EW, Truman B, Shintani A, Thomason JW, Wheeler AP, Gordon S (2003). Monitoring sedation status over time in ICU patients: reliability and validity of the Richmond Agitation-Sedation Scale (RASS). JAMA.

[13] Ramsay MA, Savege TM, Simpson BR, Goodwin R (1974). Controlled sedation with alphaxalone-alphadolone. Br Med J.

[14] Ely EW, Margolin R, Francis J, May L, Truman B, Dittus R (2001). Evaluation of delirium in critically ill patients: validation of the Confusion Assessment Method for the Intensive Care Unit (CAM-ICU). Crit Care Med.

[15] Intensive Care Society Review of Best Practice for Analgesia and Sedation in the Critical Care. 2014. http://www.ics.ac.uk/ICS/guidelines-and-standards.aspx. Accessed 25 Oct 2016.

[16] Yassin SM, Terblanche M, Yassin J, McKenzie CA (2015). A web-based survey of United Kingdom sedation practice in the intensive care unit. J Crit Care.

[17] Freilich S, Shaw MJ (2014). A census of current sedation monitoring practices in adult general intensive care units in England. JICS.

[18] O’Connor M, Bucknall T, Manias E (2010). Sedation management in Australian and New Zealand intensive care units: doctors’ and nurses’ practices and opinions. Am J Crit Care.

[19] Tanios MA, de Wit M, Epstein SK, Devlin JW (2009). Perceived barriers to the use of sedation protocols and daily sedation interruption: a multidisciplinary survey. J Crit Care.

[20] Robinson BR, Berube M, Barr J, Riker R, Gélinas C (2013). Psychometric analysis of subjective sedation scales in critically ill adults. Crit Care Med.

[21] Dellinger RP, Levy MM, Rhodes A, Annane D, Gerlach H, Opal SM (2013). Surviving Sepsis Campaign: international guidelines for management of severe sepsis and septic shock: 2012. Crit Care Med.

[22] Department of Health. Saving Lives: reducing infection, delivering clean and safe care. London: Department of Health; 21 June 2007. http://webarchive.nationalarchives.gov.uk/+/www.dh.gov.uk/en/Publicationsandstatistics/Publications/PublicationsPolicyAndGuidance/DH_078134. Accessed 25 Oct 2016.

[23] Kress JP, Pohlman AS, O’Connor MF, Hall JB (2000). Daily interruption of sedative infusions in critically ill patients undergoing mechanical ventilation. N Engl J Med.

[24] Schweickert WD, Gehlbach BK, Pohlman AS, Hall JB, Kress JP (2004). Daily interruption of sedative infusions and complications of critical illness in mechanically ventilated patients. Crit Care Med.

[25] Girard TD, Kress JP, Fuchs BD, Thomason JW, Schweickert WD, Pun BT (2008). Efficacy and safety of a paired sedation and ventilator weaning protocol for mechanically ventilated patients in intensive care (Awakening and Breathing Controlled trial): a randomised controlled trial. Lancet.

