# Supplementary material for: National survey and point prevalence study of sedation practice in UK critical care
Source: Crit Care. 2016 Oct 27;20:355. doi: 10.1186/s13054-016-1532-x (PMC5084331; doi:10.1186/s13054-016-1532-x)
Supplement: Additional file 1: Figure S1. — National survey tool. National survey instrument of sedation practice used in the present study. (PDF 226 kb) [file 13054_2016_1532_MOESM1_ESM.pdf]

icnarc intensive care national audit & research centre

Napier House
24 High Holborn
London WC1V 6AZ
tel +44 (0)20 7831 6878
fax +44 (0)20 7831 6879
email icnarc@icnarc.org

Sedation practice in UK critical care

This questionnaire asks about current sedation/analgesic practice in your unit and is part of a study that has been commissioned by the NIHR Health Technology Assessment Programme (Project: 13/159/01). The overall aim of the study is to provide up-to-date baseline data on current practice in the use of sedative agents in UK adult, general critical care units to inform evidence synthesis and potential future primary research.

Options for completing the questionnaire are:

- **On paper** – post to:
  ICNARC, Napier House, 24 High Holborn, London WC1V 6AZ
- **Electronically** – email completed questionnaire to ctu@icnarc.org
- **Online** – click on this link https://www.surveymonkey.com/s/sedationquestionnaire

Hospital name:

Name (person completing questionnaire):

Job title *(please tick)*:

Clinical Lead / Director	☐
Consultant	☐
Matron	☐
Sister / Charge Nurse	☐
Research Nurse	☐
Other *(please state)*	☐

NHS
National Institute for Health Research

1. Do you have a written sedation or sedation/analgesia protocol? *(tick one box)*

Yes	☐
No	☐

please provide a copy

*1a. **If yes to 1**, do you routinely audit compliance with the sedation or sedation/analgesia protocol? (tick one box)*

Yes	☐
No	☐

*1b. **If yes to 1a**, what is the approximate level of compliance with the sedation or sedation/analgesia protocol in your unit (e.g. 60%)?*

%

2. Is a sedation scale/score routinely used in your unit? *(tick one box)*

Yes	☐
No	☐

*2a. **If yes to 2**, which sedation scale/score is routinely used in your unit? (tick one box)*

Ramsay Sedation Scale	☐
Richmond Agitation Sedation Scale (RASS)	☐
Bispectral Index Score (BIS)	☐
Bloomsbury Sedation Scale	☐
Addenbrooke's Sedation Scale	☐
Other *(please state)*	☐

3. Is a sedation hold considered daily for patients in your unit? *(tick one box)*

Yes	☐
No	☐

*3a. **If yes to 3**, do you routinely audit compliance – i.e. that a sedation hold is considered daily for patients in your unit? (tick one box)*

Yes	☐
No	☐

*3b. **If yes to 3a**, what is the approximate level of compliance with consideration of a sedation hold (e.g. 60%)?*

%

4. Do you screen patients daily for delirium? *(tick one box)*

Yes	☐
No	☐

4a. ***If yes to 4**, which tool do you use to screen for delirium?* *(tick one box)*

Confusion Assessment Method for the ICU (CAM-ICU)	☐
Delirium Detection Score (DDS)	☐
Nursing Delirium Screening Scale (Nu-DESC)	☐
Other *(please state)*	☐

5. Do you use a continuous infusion of a neuromuscular blocking agent for sedated and ventilated patients (apart from short-term use for transfer/intubation)? *(tick one box)*

Very frequently	Frequently	Occasionally	Rarely	Never
☐	☐	☐	☐	☐

*5a. **If you do use neuromuscular blocking agent for a patient**, do you routinely monitor the depth, e.g. via Train-of-Four?* *(tick one box)*

Yes	☐
No	☐

6. How important is cost in determining your choice of agent(s) for sedation/analgesia? *(tick one box)*

Very important	Important	Neither important nor unimportant	Unimportant	Not at all important
☐	☐	☐	☐	☐

7. How important is the *expected duration* for sedation/analgesia in determining your choice of agent(s), i.e. short vs. long acting agents? *(tick one box)*

Very important	Important	Neither important nor unimportant	Unimportant	Not at all important
☐	☐	☐	☐	☐

8. For patients who require sedation, please indicate the frequency of use (bolus or infusion) of each sedative in your unit *(tick one box for each agent)*

	Very frequently	Frequently	Occasionally	Rarely	Never
Propofol	☐	☐	☐	☐	☐
Midazolam	☐	☐	☐	☐	☐
Diazepam	☐	☐	☐	☐	☐
Lorazepam	☐	☐	☐	☐	☐
Clonidine	☐	☐	☐	☐	☐
Dexmedetomidine	☐	☐	☐	☐	☐
Haloperidol	☐	☐	☐	☐	☐
Atypical psychotic for ***sedative*** purposes only	☐	☐	☐	☐	☐
Other *(please state)*	☐	☐	☐	☐	☐

*8a. Which sedative agent is generally your **first choice** for patients who require sedation?*

9. Please indicate how sedative agent(s) are used in your unit
(tick one box for each option)

	Very frequently	Frequently	Occasionally	Rarely	Never
Single sedative agent	☐	☐	☐	☐	☐
Sedative(s) in combination with one or more analgesic agents	☐	☐	☐	☐	☐
Multiple sedatives together	☐	☐	☐	☐	☐

*9a. Which of the above is your **first choice** method for patients who require sedation?*

10. For each analgesic agent listed below, please indicate the frequency of use (bolus or infusion) in your unit *(tick one box for each agent)*

	Very frequently	Frequently	Occasionally	Rarely	Never
Morphine	☐	☐	☐	☐	☐
Fentanyl	☐	☐	☐	☐	☐
Alfentanil	☐	☐	☐	☐	☐
Remifentanil	☐	☐	☐	☐	☐
Ketamine	☐	☐	☐	☐	☐
Other *(please state)*	☐	☐	☐	☐	☐

*10a. Which analgesic agent is generally your **first choice** agent for patients who require analgesia?*

THANK YOU FOR YOUR TIME
