# Supplementary material for: National survey and point prevalence study of sedation practice in UK critical care
Source: Crit Care. 2016 Oct 27;20:355. doi: 10.1186/s13054-016-1532-x (PMC5084331; doi:10.1186/s13054-016-1532-x)
Supplement: Additional file 2: Figure S2. — Point prevalence study data collection form. PPS data collection form used to collect data on all admissions in critical care units participating in the PPS on the study day. (PDF 145 kb) [file 13054_2016_1532_MOESM2_ESM.pdf]

icnarc intensive care national audit & research centre

Napier House
24 High Holborn
London WC1V 6AZ
tel +44 (0)20 7831 6878
fax +44 (0)20 7831 6879
email icnarc@icnarc.org

Sedation practice in critical care - point prevalence study

Please complete for each patient in your unit at 14:00 on 11 December 2013

HOSPITAL DETAILS

Hospital name:

PATIENT DETAILS

CMP Admission number:	NHS number:	
Has the patient received a sedative/analgesic agent in last 24 hours?	Yes ☐ No ☐	*(**If YES,** please complete the rest of the form)*

SEDATION SCORE at **14:00 on 11 December**, or most recent in last 24 hours, while ***not*** on sedation hold

Instrument:<br>(e.g. Ramsay, RASS)	Score:<br>(while not on sedation hold)
Has the patient been considered for a 'sedation hold' in the last 24 hours?	Yes ☐ No ☐
Has the patient been on a 'sedation hold' in the last 24 hours?	Yes ☐ No ☐

VENTILATION during the period: **14:00 on 10 December to 13:59 on 11 December**

Neuromuscular blocking agent: Yes ☐ No ☐<br>(*except* for transfer and/or intubation)	Ventilated via a tracheostomy? Yes ☐ No ☐

SEDATIVE AGENTS (please refer to guidance notes)

Continuous infusions received during the period: **14:00 on 10 December to 13:59 on 11 December**

	Name of sedative agent	Highest dose (mg/hour)	Route (e.g. IV)	Total dose (mg)	For delirium?
1					Yes☐ No☐
2					Yes☐ No☐
3					Yes☐ No☐

Boluses received during the period: **14:00 on 10 December to 13:59 on 11 December**

	Name of sedative	Route (e.g. IV)	Total dose (mg)	For delirium?
1				Yes☐ No☐
2				Yes☐ No☐
3				Yes☐ No☐

ANALGESIC AGENTS

Continuous infusions received during the period: **14:00 on 10 December to 13:59 on 11 December**

	Name of analgesic agent	Highest dose (mg/hour)	Route (e.g. IV, epidural)	Total dose (mg)
1				
2				
3				

Boluses received during the period: **14:00 on 10 December to 13:59 on 11 December**

	Name of analgesic agent	Route (e.g. IV, epidural)	Total dose (mg)
1			
2			
3			

Return completed forms either by fax to 020 7831 6879, or by uploading to File Exchange (see instructions)

Guidance

SEDATION SCORE

If no sedation score (while not on sedation hold) in the last 24-hours, please enter “Not done” in the **Sedation score** field.

SEDATIVE AGENTS

Do ***not*** include sedative agents given for night-time sedation or to facilitate sleep.

Continuous infusion

- **Highest dose** – please record the highest dose of sedative agent given during periods of stability (i.e. not during a procedure that necessitated increased sedation)

- **For delirium** – agents given to manage delirium (e.g. Haloperidol) should be included under Sedative agents and the **Yes** box ticked to indicate that the primary reason was to manage delirium. If the primary reason for administration was sedation, the **No** box should be ticked.

For any queries on Wednesday 11 December 2013, please call **020 7269 9295**, otherwise call the ICNARC CTU on **020 7269 9277**.
