# Supplementary material for: National survey and point prevalence study of sedation practice in UK critical care
Source: Crit Care. 2016 Oct 27;20:355. doi: 10.1186/s13054-016-1532-x (PMC5084331; doi:10.1186/s13054-016-1532-x)
Supplement: Additional file 3: Table S1. — National survey: unit characteristics by response. (PDF 69 kb) [file 13054_2016_1532_MOESM3_ESM.pdf]

Table S1 National survey – unit characteristics by response

Characteristic	Questionnaire received, n (%)	
	Yes (n=214)	No (n=21)
Country:		
England	175 (92.6)	14 (7.4)
Scotland	20 (90.9)	2 (9.1)
Wales	10 (66.7)	5 (33.3)
Northern Ireland	9 (100)	0 (0)
Hospital type:		
University	66 (89.2)	8 (10.8)
University affiliated	40 (95.2)	2 (4.8)
Non-university	108 (90.8)	11 (9.2)
Critical care unit size:		
1 to 6 beds	32 (88.9)	4 (11.1)
7 to 10 beds	79 (88.8)	10 (11.2)
11 or more beds	103 (93.6)	7 (6.4)
Participates in the Case Mix Programme:		
Yes	185 (90.7)	19 (9.3)
No[a]	29 (93.6)	2 (6.5)

[a] Includes all critical care units in Scotland, which participate in the Scottish Intensive Care Society Audit Group audit
