# Supplementary material for: National survey and point prevalence study of sedation practice in UK critical care
Source: Crit Care. 2016 Oct 27;20:355. doi: 10.1186/s13054-016-1532-x (PMC5084331; doi:10.1186/s13054-016-1532-x)
Supplement: Additional file 4: Table S2. — National survey: sedation scale score in use reported by units. (PDF 61 kb) [file 13054_2016_1532_MOESM4_ESM.pdf]

Table S2 National survey – sedation scale/score in use reported by units

| Sedation scale/score | Units, n (%) |
|---|---|
| Richmond Agitation Sedation Scale | 130 (64.7) |
| Ramsay Sedation Scale | 50 (24.9) |
| Riker Sedation Agitation Scale | 7 (3.5) |
| Bloomsbury Sedation Scale | 3 (1.0) |
| Motor Activity Assessment Scale | 1 (0.5) |
| Modified Richmond Agitation Sedation Scale | 1 (0.5) |
| Modified Ramsay Sedation Scale | 6 (3.0) |
| Local scale/score | 2 (1.0) |
| Not reported | 1 (0.5) |
