# Supplementary material for: National survey and point prevalence study of sedation practice in UK critical care
Source: Crit Care. 2016 Oct 27;20:355. doi: 10.1186/s13054-016-1532-x (PMC5084331; doi:10.1186/s13054-016-1532-x)
Supplement: Additional file 5: Table S3. — National survey: first-choice sedative agent reported by units. (PDF 58 kb) [file 13054_2016_1532_MOESM5_ESM.pdf]

Table S3 National survey – first choice sedative agent reported by units

Sedative agent	Units, n (%)[a]
Propofol	189 (88.3)
Midazolam	13 (6.1)
Not reported	15 (7.0)

[a] Three units reported both propofol and midazolam as their first choice
