# Supplementary material for: National survey and point prevalence study of sedation practice in UK critical care
Source: Crit Care. 2016 Oct 27;20:355. doi: 10.1186/s13054-016-1532-x (PMC5084331; doi:10.1186/s13054-016-1532-x)
Supplement: Additional file 6: Table S4. — National survey: first-choice analgesic agent reported by units. (PDF 61 kb) [file 13054_2016_1532_MOESM6_ESM.pdf]

Table S4 National survey – first choice analgesic agent reported by units

**Analgesic agent**	**Units, n (%)[a]**
Alfentanil	85 (39.7)
Fentanyl	56 (26.2)
Morphine	42 (19.6)
Remifentanil	32 (15.0)
Paracetamol	1 (0.5)
Not reported	7 (3.3)

[a] Five units reported both morphine and alfentanil as their first choice; two units reported both morphine and remifentanil as their first choice; one unit reported both morphine and fentanyl as their first choice; one unit reported both alfentanil and remifentanil as their first choice
