# Supplementary material for: National survey and point prevalence study of sedation practice in UK critical care
Source: Crit Care. 2016 Oct 27;20:355. doi: 10.1186/s13054-016-1532-x (PMC5084331; doi:10.1186/s13054-016-1532-x)
Supplement: Additional file 7: Table S5. — National survey: first choice of sedative analgesic delivery regimen reported by units. (PDF 59 kb) [file 13054_2016_1532_MOESM7_ESM.pdf]

Table S5 National survey – first choice of sedative/analgesic delivery regimen reported by units

**Sedative/analgesic delivery regimen**	**Units, n (%)**
Single sedative agent	9 (4.2)
Sedative(s) in combination with one or more analgesic agents	177 (82.7)
Multiple sedatives together	2 (0.9)
Not reported	26 (12.1)
