# Supplementary material for: National survey and point prevalence study of sedation practice in UK critical care
Source: Crit Care. 2016 Oct 27;20:355. doi: 10.1186/s13054-016-1532-x (PMC5084331; doi:10.1186/s13054-016-1532-x)
Supplement: Additional file 8: Table S6. — National survey: factors determining the choice of sedative/analgesic agents reported by units. (PDF 61 kb) [file 13054_2016_1532_MOESM8_ESM.pdf]

Table S6 National survey – factors determining the choice of sedative/analgesic agents reported by units

	Reported importance, *n (%)*			
**Factor**	**Very important/ Important**	**Neither important nor unimportant**	**Unimportant/ Not at all important**	**Not reported**
Cost	102 (47.7)	79 (36.9)	31 (14.5)	2 (0.9)
Expected duration for sedation and/or analgesia	176 (82.2)	18 (8.4)	18 (8.4)	2 (0.9)
