# Supplementary material for: National survey and point prevalence study of sedation practice in UK critical care
Source: Crit Care. 2016 Oct 27;20:355. doi: 10.1186/s13054-016-1532-x (PMC5084331; doi:10.1186/s13054-016-1532-x)
Supplement: Additional file 9: Table S7. — Characteristics of units in the Case Mix Programme at the time of the point prevalence study (1400 on 11 December 2013) by units that did and did not participate in the point prevalence study. (PDF 10 kb) [file 13054_2016_1532_MOESM9_ESM.pdf]

Table S7 Characteristics of units in the Case Mix Programme at the time of the point prevalence study (14.00 on 11 December 2013) by units that did and did not participate in the point prevalence study

	**Unit participated in the point prevalence study, n (%)**	
**Characteristic**	**Yes (n=50)**	**No (n=133)**
Country:		
England	46 (92.0)	118 (88.7)
Wales	1 (2.0)	10 (7.5)
Northern Ireland	3 (6.0)	5 (3.8)
Hospital type:		
University	12 (24.0)	37 (27.8)
University affiliated	14 (28.0)	21 (15.8)
Non-university	24 (48.0)	75 (56.4)
Critical care unit size:		
1 to 6 beds	8 (16.0)	17 (12.8)
7 to 10 beds	15 (30.0)	57 (42.9)
11 or more beds	27 (54.0)	59 (44.4)
Proportion of surgical admissions:		
40% or less	32 (64.0)	80 (60.2)
Greater than 40%	18 (36.0)	53 (39.8)
