# Supplementary material for: National survey and point prevalence study of sedation practice in UK critical care
Source: Crit Care. 2016 Oct 27;20:355. doi: 10.1186/s13054-016-1532-x (PMC5084331; doi:10.1186/s13054-016-1532-x)
Supplement: Additional file 10: Table S8. — Characteristics of patients in Case Mix Programme units at the time of the point prevalence study (1400 on 11 December 2013) by units that did and did not participate in the point prevalence study. (PDF 80 kb) [file 13054_2016_1532_MOESM10_ESM.pdf]

Table S8 Characteristics of patients in Case Mix Programme units at the time of the point prevalence study (14.00 on 11 December 2013) by units that did and did not participate in the point prevalence study

Characteristic[a]	Unit participated in the point prevalence study Yes (n=50)	Unit participated in the point prevalence study No (n=133)
Patients, *n*	516	1,296
Age, *mean (SD)*	61.7 (15.6)	63.2 (15.9)
Gender, *n (%)*:		
Female	224 (43.4)	571 (44.1)
Male	292 (56.6)	725 (55.9)
Ethnicity, *n (%)*:		
White	475 (94.3)	1,175 (93.0)
Black or Black British	12 (2.4)	32 (2.5)
Asian or Asian British	11 (2.2)	34 (2.7)
Mixed	2 (0.4)	9 (0.7)
Other ethnic group	4 (0.8)	13 (1.0)
Severe co-morbidities, *n (%)*:		
Liver co-morbidity	13 (2.5)	44 (3.4)
Renal co-morbidity	5 (1.0)	28 (2.2)
Respiratory co-morbidity	12 (2.3)	45 (3.5)
Cardiovascular co-morbidity	7 (1.4)	28 (2.2)
Haematological malignancy	7 (1.4)	26 (2.0)
Metastatic disease	12 (2.3)	27 (2.1)
Immunosuppression	50 (9.7)	96 (7.4)
Surgical status, *n (%)*:		
Elective/scheduled	70 (13.6)	157 (12.1)
Emergency/urgent	82 (15.9)	236 (18.2)
Non-surgical	364 (70.5)	903 (69.7)
Primary/secondary reason for admission, *n (%)*:		
Admissions following trauma	62 (12.0)	177 (13.7)
Admissions with neurological condition	46 (9.0)	109 (8.5)
ICNARC Physiology Score, *mean (SD)*	20.2 (8.7)	20.2 (8.7)
ICNARC model (2013 recalibration) predicted risk of death, *median (IQR)*	0.23 (0.08, 0.44)	0.24 (0.08, 0.45)
APACHE II Acute Physiology Score, *mean (SD)*	13.1 (5.5)	13.1 (5.8)
APACHE II Score, *mean (SD)*	17.4 (6.1)	17.7 (6.5)
APACHE II (2013 recalibration) predicted risk of death, *median (IQR)*	0.22 (0.10, 0.38)	0.22 (0.10, 0.38)
Mechanical ventilation – 1st 24 hours, *n (%)*	306 (59.3)	711 (55.0)
Time (days) since admission to critical care, *mean (SD)*	9.0 (13.3)	8.3 (10.8)

SD: standard deviation; ICNARC: Intensive Care National Audit & Research Centre; IQR: interquartile range; APACHE: Acute Physiology And Chronic Health Evaluation

[a] Case mix was assessed by: age; sex; ethnicity; severe co-morbidities; surgical status; primary/secondary reason for unit admission; acute severity of illness; and time since admission to the critical care unit
