# Supplementary material for: National survey and point prevalence study of sedation practice in UK critical care
Source: Crit Care. 2016 Oct 27;20:355. doi: 10.1186/s13054-016-1532-x (PMC5084331; doi:10.1186/s13054-016-1532-x)
Supplement: Additional file 11: Table S9. — Outcome and length of stay for patients in Case Mix Programme units on the day of the point prevalence study (1400 on 11 December 2013) by units that did and did not participate in the point prevalence study. (PDF 71 kb) [file 13054_2016_1532_MOESM11_ESM.pdf]

Table S9 Outcome and length of stay for patients in Case Mix Programme units on the day of the point prevalence study (14.00 on 11 December 2013) by units that did and did not participate in the point prevalence study

	Participated in the point prevalence study	
**Outcome [a] and length of stay [b]**	**Yes (n=50)**	**No (n=133)**
Number of patients	516	1,296
Deaths – critical care, *n (%)*	80 (15.5)	199 (15.4)
Deaths – acute hospital[c], *n (%)*	121 (25.0)	331 (26.9)
LOS (days) – critical care, *mean (SD)*		
Survivors	17.3 (19.6)	16.2 (18.8)
Non-survivors	16.7 (18.0)	18.7 (21.1)
LOS (days) – critical care, *median (IQR)*		
Survivors	9.6 (3.9, 23.3)	9.2 (4.0, 21.7)
Non-survivors	10.4 (4.7, 24.6)	12.4 (5.0, 23.9)
LOS (days) – acute hospital, *mean (SD)*		
Survivors	42.1 (42.3)	41.7 (55.4)
Non-survivors	31.0 (32.5)	31.4 (30.2)
LOS (days) – acute hospital, *median (IQR)*		
Survivors	28 (12, 60)	28 (13, 55)
Non-survivors	23 (9, 38)	21 (10, 45)

LOS: length of stay; SD: standard deviation; IQR: interquartile range

[a] Outcome was assessed by mortality at unit and acute hospital discharge

[b] Length of stay was assessed both in the unit and for the total stay in an acute hospital and was stratified by outcome

[c] Acute hospital outcomes missing for 96 patients (32 from units participating in the point prevalence study, 64 from units not participating in the point prevalence study) at the point of data lock
