# Supplementary material for: National survey and point prevalence study of sedation practice in UK critical care
Source: Crit Care. 2016 Oct 27;20:355. doi: 10.1186/s13054-016-1532-x (PMC5084331; doi:10.1186/s13054-016-1532-x)
Supplement: Additional file 12: Table S10. — Sedation score/scale in use reported in the national survey by units that did and did not participate in the point prevalence study. (PDF 7 kb) [file 13054_2016_1532_MOESM12_ESM.pdf]

Table S10 Sedation score/scale in use reported in the national survey by units that did and did not participate in the point prevalence study

Sedation scale/score[a]	Unit participated in the point prevalence study, n (%)	
	**Yes (n=51)**	**No (n=163)**
Richmond Agitation Sedation Scale	34 (68.0)	96 (63.6)
Ramsay Sedation Scale	12 (24.0)	38 (25.2)
Riker Sedation Agitation Scale	2 (4.0)	5 (3.3)
Bloomsbury Sedation Scale	0 (0)	3 (2.0)
Other	2 (4.0)	8 (5.3)
Not reported	0 (0)	1 (0.7)

[a] 13 units (one participating in point prevalence study, 12 not participating in point prevalence study) reported not routinely using a sedation scale/score
