# Supplementary material for: National survey and point prevalence study of sedation practice in UK critical care
Source: Crit Care. 2016 Oct 27;20:355. doi: 10.1186/s13054-016-1532-x (PMC5084331; doi:10.1186/s13054-016-1532-x)
Supplement: Additional file 13: Table S11. — Sedative agents, analgesic agents and sedative/analgesic delivery regimens and their reported frequency of use in the national survey by units that did and did not participate in the point prevalence study. (PDF 86 kb) [file 13054_2016_1532_MOESM13_ESM.pdf]

Table S11 Sedative agents, analgesic agents and sedative/analgesic delivery regimens and their reported frequency of use in the national survey by units that did and did not participate in the point prevalence study

	**Reported frequency of use, *n (%)***							
	**Very frequently/ Frequently**		**Occasionally/Rarely**		**Never**		**Not reported**	
	**PPS unit**[a]	**Non-PPS unit**[b]	**PPS unit**[a]	**Non-PPS unit**[b]	**PPS unit**[a]	**Non-PPS unit**[b]	**PPS unit**[a]	**Non-PPS unit**[b]
Sedative agent:								
Propofol	51 (100)	159 (97.5)	0 (0)	2 (1.2)	0 (0)	0 (0)	0 (0)	2 (1.2)
Midazolam	15 (29.4)	54 (33.1)	32 (62.7)	98 (60.1)	4 (7.8)	7 (4.3)	0 (0)	4 (2.5)
Diazepam	0 (0)	4 (2.5)	20 (39.2)	76 (46.6)	30 (58.8)	75 (46.0)	1 (2.0)	8 (4.9)
Lorazepam	0 (0)	2 (1.2)	27 (52.9)	93 (57.1)	23 (45.1)	60 (36.8)	1 (2.0)	8 (4.9)
Clonidine	18 (35.3)	52 (31.9)	30 (58.8)	99 (60.7)	3 (5.8)	5 (3.1)	0 (0)	7 (4.3)
Dexmedetomidine	6 (11.8)	16 (9.8)	11 (21.6)	46 (28.2)	33 (64.7)	94 (57.7)	1 (2.0)	7 (4.3)
Haloperidol	12 (23.5)	67 (41.1)	31 (60.8)	84 (51.5)	6 (11.8)	7 (4.3)	2 (3.9)	5 (3.1)
Atypical anti-psychotic[c]	5 (9.8)	10 (6.1)	19 (37.3)	75 (46.0)	23 (45.1)	66 (40.5)	4 (7.8)	12 (7.4)
Other	8 (15.7)	18 (11.0)	3 (5.9)	9 (5.5)	-	-	40 (78.4)	136 (83.4)
Analgesic agent:								
Morphine	18 (35.3)	72 (44.2)	31 (60.8)	83 (50.9)	1 (2.0)	5 (3.1)	1 (2.0)	3 (1.8)
Fentanyl	17 (33.3)	60 (36.8)	20 (39.2)	69 (42.3)	14 (27.5)	27 (16.6)	0 (0)	7 (4.3)
Alfentanil	26 (51.0)	84 (51.5)	8 (15.7)	45 (27.6)	16 (31.4)	30 (18.4)	1 (2.0)	4 (2.5)
Remifentanil	19 (37.3)	53 (32.5)	24 (47.1)	82 (50.3)	8 (15.7)	24 (14.7)	0 (0)	4 (2.5)
Ketamine	0 (0)	1 (0.6)	40 (78.4)	137 (84.1)	10 (19.6)	18 (11.0)	1 (2.0)	7 (4.3)
Other	0 (0)	3 (1.8)	0 (0)	9 (5.5)	-	-	51 (100)	151 (92.6)
Sedative/analgesic delivery regimen:								
Single sedative agent	7 (13.7)	40 (24.5)	39 (76.5)	103 (63.2)	0 (0)	4 (2.5)	5 (9.8)	16 (9.8)
Sedative(s) in combination with one or more analgesic agents	50 (98.0)	157 (96.3)	1 (2.0)	3 (1.8)	0 (0)	0 (0)	0 (0)	3 (1.8)
Multiple sedatives together	8 (15.7)	15 (9.2)	39 (76.5)	124 (76.1)	1 (2.0)	4 (2.5)	3 (5.9)	20 (12.3)

PPS – point prevalence study
[a] n=51 units
[b] n=163 units
[c] For sedative purposes only
