# Supplementary material for: National survey and point prevalence study of sedation practice in UK critical care
Source: Crit Care. 2016 Oct 27;20:355. doi: 10.1186/s13054-016-1532-x (PMC5084331; doi:10.1186/s13054-016-1532-x)
Supplement: Additional file 14: Table S12. — First choice of sedative agent reported in the national survey by units that did and did not participate in the point prevalence study. (PDF 6 kb) [file 13054_2016_1532_MOESM14_ESM.pdf]

Table S12 First choice of sedative agent reported in the national survey by units that did and did not participate in the point prevalence study

	**Unit participated in the point prevalence study, n (%)**	
**Sedative agent[a]**	**Yes (n=51)**	**No (n=163)**
Propofol	47 (92.2)	142 (87.1)
Midazolam	3 (5.9)	10 (6.1)
Not reported	1 (2.0)	14 (8.6)

[a] Three units (all not participating in the point prevalence study) reported both propofol and midazolam as their first choice
