# Supplementary material for: National survey and point prevalence study of sedation practice in UK critical care
Source: Crit Care. 2016 Oct 27;20:355. doi: 10.1186/s13054-016-1532-x (PMC5084331; doi:10.1186/s13054-016-1532-x)
Supplement: Additional file 15: Table S13. — First choice of analgesic agent reported in the national survey by units that did and did not participate in the point prevalence study. (PDF 7 kb) [file 13054_2016_1532_MOESM15_ESM.pdf]

Table S13 First choice of analgesic agent reported in the national survey by units that did and did not participate in the point prevalence study

	**Unit participated in the point prevalence study, n (%)**	
**Analgesic agent[a]**	**Yes (n=51)**	**No (n=163)**
Morphine	10 (19.6)	32 (19.6)
Fentanyl	13 (25.5)	43 (26.4)
Alfentanil	21 (41.2)	64 (39.3)
Remifentanil	8 (15.7)	24 (14.7)
Paracetamol	0 (0)	1 (0.6)
Not reported	1 (2.0)	6 (3.7)

[a] Five units (one participating in point prevalence study, four not participating in point prevalence study) reported both morphine and alfentanil as their first choice; two units (one participating in point prevalence study, one not participating in point prevalence study) reported both morphine and remifentanil as their first choice; one unit (not participating in point prevalence study) reported both morphine and fentanyl as their first choice; one unit (not participating in point prevalence study) reported both alfentanil and remifentanil as their first choice
