# Supplementary material for: National survey and point prevalence study of sedation practice in UK critical care
Source: Crit Care. 2016 Oct 27;20:355. doi: 10.1186/s13054-016-1532-x (PMC5084331; doi:10.1186/s13054-016-1532-x)
Supplement: Additional file 16: Table S14. — First choice for delivery of sedation and analgesia in the national survey by units that did and did not participate in the point prevalence study. (PDF 6 kb) [file 13054_2016_1532_MOESM16_ESM.pdf]

Table S14 First choice for delivery of sedation and analgesia in the national survey by units that did and did not participate in the point prevalence study

	**Unit participated in the point prevalence study, n (%)**	
**Sedative/analgesic delivery regimen**	**Yes (n=51)**	**No (n=163)**
Single sedative agent	1 (2.0)	8 (4.9)
Sedative(s) in combination with one or more analgesic agents	43 (84.3)	134 (82.2)
Multiple sedatives together	1 (2.0)	1 (0.6)
Not reported	6 (11.8)	20 (12.3)
