# Supplementary material for: National survey and point prevalence study of sedation practice in UK critical care
Source: Crit Care. 2016 Oct 27;20:355. doi: 10.1186/s13054-016-1532-x (PMC5084331; doi:10.1186/s13054-016-1532-x)
Supplement: Additional file 17: Table S15. — Point prevalence study: characteristics, outcomes and length of stay of sedated and non-sedated patients with validated Case Mix Programme data (n = 50 units). (PDF 86 kb) [file 13054_2016_1532_MOESM17_ESM.pdf]

Table S15 Point prevalence study – characteristics, outcomes and length of stay of sedated and non-sedated patients with validated Case Mix Programme data (n=50 units)

	Sedated (n=230)	Non-sedated (n=286)
**Characteristic**[a]		
Age, *mean (SD)*	59.3 (16.3)	63.6 (14.8)
Gender, *n (%)*		
Female	100 (43.5)	124 (43.4)
Male	130 (56.5)	162 (56.6)
Ethnicity, *n (%)*:		
White	214 (94.7)	261 (93.9)
Black or Black British	4 (1.8)	8 (2.9)
Asian or Asian British	6 (2.7)	5 (1.8)
Mixed	2 (0.9)	0 (0.0)
Other ethnic group	0 (0.0)	4 (1.4)
Severe co-morbidities, *n (%)*:		
Liver co-morbidity	10 (4.3)	3 (1.0)
Renal co-morbidity	3 (1.3)	2 (0.7)
Respiratory co-morbidity	5 (2.2)	7 (2.4)
Cardiovascular co-morbidity	2 (0.9)	5 (1.7)
Haematological malignancy	3 (1.3)	4 (1.4)
Metastatic disease	6 (2.6)	6 (2.1)
Immunosuppression	22 (9.5)	28 (9.8)
Surgical status, *n (%)*:		
Elective/scheduled	24 (10.4)	46 (16.1)
Emergency/urgent	44 (19.1)	38 (13.3)
Non-surgical	162 (70.4)	202 (70.6)
Primary/secondary reason for admission, *n (%)*:		
Admissions following trauma	31 (13.5)	31 (10.8)
Admissions with neurological condition	25 (10.9)	21 (7.5)
ICNARC Physiology Score, *mean (SD)*	22.7 (8.7)	18.1 (8.3)
ICNARC model (2013 recalibration) predicted risk of death, *median (IQR)*	0.30 (0.12, 0.52)	0.20 (0.05, 0.38)
APACHE II Acute Physiology Score, *mean (SD)*	13.6 (5.5)	12.7 (5.5)
APACHE II Score, *mean (SD)*	17.7 (6.2)	17.2 (6.0)
APACHE II (2013 recalibration) predicted risk of death, *median (IQR)*	0.23 (0.12, 0.41)	0.20 (0.09, 0.35)
Mechanical ventilation – 1st 24 hours, *n (%)*	181 (78.4)	125 (43.7)
Time (days) since admission to critical care, *mean (SD)*	6.9 (9.5)	10.6 (15.5)
**Outcome**[b] **and length of stay**[c]		
Deaths – critical care, *n (%)*	58 (25.2)	22 (7.7)
Deaths – acute hospital[d], *n (%)*	74 (33.6)	47 (17.8)
LOS (days) – critical care, *mean (SD)*		
Survivors	17.6 (16.4)	17.1 (21.5)
Non-survivors	17.0 (18.6)	16.0 (16.9)
LOS (days) – critical care, *median (IQR)*		
Survivors	12.7 (5.8, 25.3)	8.0 (3.4, 21.2)
Non-survivors	10.4 (6.1, 23.5)	10.6 (1.8, 25.4)
LOS (days) – acute hospital, *mean (SD)*		
Survivors	43.7 (35.2)	41.0 (46.5)
Non-survivors	24.9 (22.8)	40.5 (42.2)
LOS (days) – acute hospital, *median (IQR)*		
Survivors	33 (16, 62)	24 (11, 59)
Non-survivors	19 (9, 32)	27 (9, 58)

SD: standard deviation; ICNARC: Intensive Care National Audit & Research Centre; IQR: interquartile range; APACHE: Acute Physiology And Chronic Health Evaluation; LOS: length of stay

[a] Case mix was assessed by: age; sex; ethnicity; severe co-morbidities; surgical status; primary/secondary reason for unit admission; acute severity of illness; and time since admission to the critical care unit
[b] Outcome was assessed by mortality at unit and acute hospital discharge
[c] Length of stay was assessed both in the unit and for the total stay in an acute hospital and was stratified by outcome
[d] Acute hospital outcomes missing for 32 patients (10 sedated, 22 non-sedated) at the point of data lock
